# Exploring the prediction model and core genes for coronary artery disease in non-obese steatotic liver disease patients

**DOI:** 10.3389/fmed.2026.1709412

**Published:** 2026-02-09

**Authors:** Yue Zhang, Xiangjian Song, Mei Han, Xiaoyun Gao, Shuanglin Han, Pujun Gao

**Affiliations:** 1 Department of Hepatology, The First Hospital of Jilin University, Changchun, Jilin, China; 2 Department of Gastroenterology, The Second Hospital of Dalian Medical University, Dalian, China; 3 Department of Geriatric, The Second Hospital of Dalian Medical University, Dalian, China

**Keywords:** core genes, coronary artery disease, machine learning, metabolic dysfunction-associated steatotic liver disease, nomogram, prediction model

## Abstract

**Backgrounds and aims:**

Non-obese steatotic liver disease (SLD) refers to a metabolic disorder characterized by ectopic fat deposition in the liver, but without increased subcutaneous adipose tissue and normal body mass index (BMI) in patients. Emerging evidence indicates that non-obese SLD is associated with coronary artery disease (CAD). However, the mechanisms underlying their mutual relationship remain undefined.

**Methods:**

We retrospectively analyzed 8,722 subjects and constructed a prediction model for diagnosing CAD in non-obese SLD patients. Then, public datasets from the Gene Expression Omnibus (GEO) were retrieved for further bioinformatics analysis, and machine learning algorithms were used to screen candidate core genes.

**Results:**

Through the analysis of clinical data, we found that the risk of CAD in non-obese SLD patients was significantly higher than that in obese SLD patients and individuals without SLD. We constructed a nomogram for predicting CAD in non-obese SLD patients, and the area under the curve for training and validation sets was 0.846 and 0.732, respectively. We analyzed the non-obese SLD dataset (GSE89632) and CAD dataset (GSE113079) and overlapped the differentially expressed genes (DEGs) in these two datasets. We found that there were 28 overlapping upregulated DEGs and 66 overlapping downregulated DEGs. The protein–protein interaction network generated a 94-edge network, and the top 40 hub genes were selected using the maximal clique centrality algorithm. The candidate core genes, including HNF4A and LTBP4, were screened based on machine learning algorithms. The receiver operating characteristic results showed that these two genes have considerable diagnostic value for non-obese SLD and CAD.

**Conclusion:**

We found a close correlation between non-obese SLD and CAD. Our study developed a novel diagnostic model to predict CAD in non-obese SLD patients with promising predictive performance. In addition, through comprehensive bioinformatics analysis and machine learning algorithms, two key core genes, HNF4A and LTBP4, were identified to be associated with both non-obese SLD and CAD.

## 1 Introduction

Metabolic dysfunction-associated steatotic liver disease (MASLD) is one of the most common liver diseases and affects approximately 25% of the global population, which is the most common type of steatotic liver disease (SLD) (1, 2). Traditionally, MASLD is considered to be associated with overweight or obesity. Nonetheless, SLD has also been increasingly reported in normal-weight individuals. Non-obese SLD is considered ‘metabolically unhealthy normal weight’ SLD, characterized by no significant increase in subcutaneous fat accompanied by ectopic fat deposition in the liver (3, 4). The pathogenesis of non-obese SLD is different from conventional MASLD due to different metabolic and gut microbiota profiles (5, 6).

Cardiovascular diseases are the predominant global cause of death, with an estimated 18 million people dying from these diseases per year, which accounts for 31% of total deaths (7). Coronary artery disease (CAD) refers to the accumulation of lipids and immune cells in the subendothelial layer of the coronary arteries, or atherosclerosis, which is the primary cause of death from cardiovascular diseases, accounting for nearly 45% of such cases (8–10). Patients with MASLD are associated with a higher risk of cardiovascular events (11, 12). A recent large-scale retrospective cohort study demonstrated that cardiovascular diseases were one of the leading causes of mortality in patients with non-cirrhotic steatotic liver disease (13). In previous studies that compared obese MASLD patients and non-obese SLD patients, non-obese SLD individuals may lead to a more severe prognosis, including a higher risk of cardiovascular disease (14), severe liver disease (15, 16), hypertension, dyslipidemia, metabolic syndromes (17, 18), and all-cause mortality (19). However, other studies have also shown controversial conclusions. For example, some previous research showed that non-obese SLD had a lower prevalence of cardiovascular events (20, 21), hypertension, diabetes (DM), and metabolic syndromes (22) compared to obese MASLD patients. These discrepancies indicate that obese MASLD and non-obese SLD differ significantly in their clinical associations, particularly regarding cardiovascular events, though the exact underlying pathogenesis remains unclear.

In order to further study the relationship between non-obese SLD and CAD, we recruited subjects who completed coronary computed tomography angiography (CCTA) screening to assess coronary atherosclerosis and liver imaging to assess liver status. In the study, we analyzed baseline clinical characteristics and metabolic parameters to identify risk factors for CAD within the non-obese SLD cohort. Subsequently, we constructed the prediction model of CAD in non-obese SLD and explored shared core genes linking the two conditions through bioinformatics analysis and machine learning.

## 2 Methods

### 2.1 Clinical data analysis

#### 2.1.1 Study population

A retrospective cross-sectional study was performed, including patients hospitalized at the Second Hospital of Dalian Medical University from January 2016 to June 2024. We collected the clinical data of patients who underwent liver imaging examinations and CCTA scans due to thoracalgia or physical examination. Existing literature indicates a high degree of concordance (>96%) between NAFLD and MASLD definitions, with several studies suggesting they may be used interchangeably in retrospective contexts (23, 24). Since a portion of our participants lacked recorded values for waist circumference, quantitative blood pressure reading or glycated hemoglobin measurements, for the diagnosis of SLD or MAFLD, we still implemented the diagnostic criteria for NAFLD: requires (a) evidence of hepatic steatosis by imaging, and (b) no causes of secondary hepatic fat accumulation such as significant alcohol consumption, use of steatogenic medication or hereditary disorders (25). The diagnostic criteria for CAD are that coronary artery stenosis reaches at least 50% under CCTA scan. Weight (in kilograms) and height (in centimeters) were objectively measured by nurses at the time of admission. SLD patients were divided into two groups according to body mass index (BMI) levels (BMI < 25 kg/m^2^ as non-obese SLD group, BMI ≥ 25 mg/m^2^ as obese MASLD group). Participants with normal ultrasound findings were defined as the healthy control group. This study was approved by the Ethics Committee of the Second Hospital of Dalian Medical University. This study used anonymous data, and informed consent was obtained before the CCTA scan.

#### 2.1.2 Collection of clinical data

Baseline characteristics of enrolled patients were collected, including demographic data (age and sex), anthropometric measurements (height and weight), and medical history (specifically hypertension and diabetes). Laboratory assessments included serum levels of apolipoprotein B/apolipoprotein A1 [ApoB/A1], albumin [Alb], alanine aminotransferase [ALT], absolute Neutrophil Count [ANC], aspartate aminotransferase [AST], creatinine [Cr], fasting blood glucose [FBG], hemoglobin [HGB], hemoglobin A1c [HbA1c], high-density lipoprotein cholesterol [HDL-C], low-density lipoprotein cholesterol [LDL-C], white blood cell [WBC], platelet count [PLT], red blood cell [RBC], total bilirubin [TBIL], total cholesterol [TC], triglyceride [TG], and uric acid [UA]. Imaging modalities included abdominal CT scan, abdominal ultrasound, and CCTA scan. Anthropometric data were collected on the day of admission, and laboratory tests were conducted after overnight fasting within 24 h after admission. BMI was calculated by dividing a patient’s weight (in kg) by the square of his height (in meters).

#### 2.1.3 Statistical analyses

Continuous variables were assessed for normality using the Shapiro–Wilk test. As the majority of the parameters did not follow a normal distribution, they were presented as medians along with interquartile ranges (25th–75th percentiles) and were compared by the Mann–Whitney U-test. Categorical variables (gender, prevalence of hypertension, DM, and CAD) were expressed as counts and percentages and analyzed by chi-square tests. Non-obese SLD patients were randomly divided into a training set and a validation set at a ratio of 3:2. After assessing for multicollinearity, multivariate stepwise logistic regression analysis was conducted to evaluate the association strength of the independent risk factors for CAD in non-obese SLD, and odds ratios (ORs) with 95% confidence intervals (CIs) were displayed. Selected variables were incorporated into a nomogram to predict the risk of CAD in non-obese SLD patients. The area under the curve (AUC) of the receiver operating characteristic (ROC) was used to assess the discriminative ability of the prediction models in both sets. Calibration curve and decision curve analysis (DCA) were performed to evaluate the accuracy and clinical utility of the prediction models. Statistical analyses were performed by SPSS (version 27) and R software (version 4.2.3). The packages used included “readr,” “MASS,” “rms,” and “pROC.” A two-sided *p*-value of < 0.05 was considered statistically significant.

### 2.2 Transcriptomics analyses

#### 2.2.1 Data collection and processing

We downloaded two microarray datasets from the Gene Expression Omnibus (GEO^1^). The GSE89632 dataset contains liver tissue gene expression profiles, including both non-obese SLD patients and obese MASLD patients (GSE89632: *n* = 6 non-obese SLD, *n* = 31 obese MASLD). GSE113079 contains peripheral blood mononuclear cells gene expression profiles, including CAD patients and healthy controls (GSE113079: *n* = 93 CAD, *n* = 48 controls). Then we normalized the raw data and calculated the average expression when multiple probes referred to the same gene symbol. Detailed dataset characteristics are provided in Supplementary Table 1.

#### 2.2.2 Identification of differentially expressed genes in CAD and non-obese SLD

We identified differentially expressed genes (DEGs) in non-obese SLD and CAD by the “limma” R package. A two-sided *p*-value of < 0.05 was used as the chosen standard for DEGs. Genes with log2 fold change (log2 FC) > 0 were considered as upregulated genes, and genes with log2 FC < 0 as downregulated genes. “randomcoloR” and “venn” packages were used to identify and show the overlap of DEGs between GSE89632 and GSE113079 datasets.

#### 2.2.3 Construction of protein–protein interaction and identification of hub genes

Overlapping DEGs were analyzed by the Search Tool for the Retrieval of Interacting Genes/Proteins (STRING) database^2^ to construct a protein–protein interaction (PPI) network. We set the parameters to “*Homo sapiens*” with a minimum interaction score threshold of 0.4. Hub DEGs were visualized by Cytoscape (Version 3.9.1) plug-in CytoHubba.^3^ The maximal clique centrality (MCC) algorithm screened out nodes with the top 40 highest scores as hub genes.

#### 2.2.4 Screening optimal core genes via machine learning

Support vector machine-recursive feature elimination (SVM-RFE), least absolute shrinkage and selection operator (LASSO), and random forest (RF) were used to identify the optimal core genes in GSE89632. SVM-RFE was implemented using the “sigFeature” and “e1071” R packages to select the most relevant gene subset and prevent overfitting, with feature precedence determined by AvgRank. The optimal parameter *λ* was determined by 10-fold cross-validation of LASSO with the “glmnet” R package. RF is a supervised machine learning method that is performed by the “randomForest” R package. In this study, genes with importance > 0 were considered as candidate genes. Subsequently, by crossing the significant genes identified by these three machine learning methods, the optimal core genes were screened out.

#### 2.2.5 Exploring the expression levels of core genes in non-obese populations

The expression levels of the optimal core genes in the datasets (GSE89632 and GSE113079) were calculated using the “ggplot2” R package. Boxplots were generated to compare core gene expression levels between the disease groups (non-obese SLD and CAD) and healthy groups. Statistical significance was assessed by the Wilcoxon test (*p* < 0.05).

#### 2.2.6 Evaluation of diagnostic efficacy

To evaluate the diagnostic efficiency of core genes for non-obese SLD and CAD, AUCs were calculated by the “pROC” R package.

#### 2.2.7 Statistical analyses

All statistical analyses and graph generation for bioinformatics studies were performed using R software (version 4.2.3). The differences between the two groups were assessed using the Wilcoxon signed-rank test. A two-sided *p*-value of < 0.05 was considered statistically significant.

## 3 Results

### 3.1 Demographic and clinical characteristics of the control, non-obese SLD, and obese MASLD group

A total of 8,722 participants who underwent both hepatic imaging examination and CCTA scan were enrolled in the analysis. Based on the liver imaging results and BMI, the participants were categorized into three groups: the control group (6,726, 77.12%), the non-obese SLD group (517, 5.92%), and the obese MASLD group (1,479, 16.96%). The clinical characteristics of this group are presented in Table 1.

**Table 1:** Demographic and clinical characteristics of participants in the control, non-obese SLD, and obese MASLD groups.

	Control (*n* = 6,726)	Non-obese SLD (n = 517)	Obese MASLD (*n* = 1,479)	*p* ^1^	*p* ^2^	*p* ^3^
Age (years)	63 (56, 70)	61 (54, 66.5)	59 (50, 66)	<0.001	<0.001	<0.001
BMI (kg/m^2^)	25.25 (23.15, 27.39)	23.92 (22.86, 24.57)	28.08 (26.61, 30.07)	<0.001	<0.001	<0.001
FBG (mmol/L)	5.57 (5.06, 6.65)	5.72 (5.10, 6.98)	6.22 (5.31, 7.64)	<0.001	<0.001	0.012
HbA1c (%)	5.80 (5.50, 6.20)	5.90 (5.60, 6.50)	5.90 (5.60, 6.30)	<0.001	<0.001	1.000
WBC (×10^9^/L)	5.90 (4.99, 7.06)	6.16 (5.18, 7.15)	6.26 (5.36, 7.40)	0.067	<0.001	0.137
ANC (×10^9^/L)	3.65 (2.92, 4.53)	3.68 (2.96, 4.63)	3.79 (3.06, 4.71)	1.000	<0.001	0.228
RBC (×10^12^/L)	4.54 (4.24, 4.86)	4.60 (4.31, 4.91)	4.75 (4.45, 5.10)	0.112	<0.001	<0.001
HGB (g/L)	137 (128, 148)	139 (131,150)	144 (134, 155)	0.12	<0.001	<0.001
PLT (×10^9^/L)	217 (184, 254)	226 (195.25, 264)	227 (195, 261)	0.001	<0.001	1.000
Urea (mmol/L)	5.80 (4.90, 6.80)	5.60 (4.71, 6.60)	5.63 (4.80, 6.60)	0.004	0.096	0.298
Cr (μmol/L)	64.30 (54.96, 76.20)	61.65 (52.30, 71.43)	64.35 (55.20, 75.85)	<0.001	1.000	<0.001
UA (μmol/L)	329.90 (274.23, 392.50)	335.49 (282.55, 400.79)	359.01 (305.98, 422.03)	0.436	<0.001	<0.001
TC (mmol/L)	4.73 (4.00, 5.48)	5.01 (4.17, 5.75)	4.80 (4.14, 5.51)	<0.001	0.017	0.009
TG (mmol/L)	1.32 (0.98, 1.85)	1.55 (1.18, 2.30)	1.70 (1.26, 2.36)	<0.001	<0.001	0.015
HDL (mmol/L)	1.20 (1.02, 1.42)	1.16 (1.01, 1.37)	1.11 (0.9, 1.29)	0.025	<0.001	<0.001
LDL (mmol/L)	2.54 (2.00, 3.08)	2.72 (2.09, 3.30)	2.58 (2.09, 3.11)	0.001	0.070	0.145
Apo B/A1	0.64 (0.53, 0.78)	0.68 (0.57, 0.81)	0.70 (0.58, 0.82)	<0.001	<0.001	0.815
ALT (IU/L)	19.71 (14.64, 27.27)	23.19 (17.41, 32.66)	27.30 (19.64, 40.71)	<0.001	<0.001	<0.001
AST (IU/L)	20.23 (16.91, 24.49)	21.44 (17.51, 26.02)	21.60 (17.99, 27.84)	<0.001	<0.001	0.497
Alb (g/L)	42.30 (39.83, 44.70)	43.07 (40.77, 45.40)	43.44 (40.99, 45.62)	<0.001	<0.001	0.201
TBIL (μmol/L)	12.64 (9.76, 16.30)	12.60 (9.69, 16.13)	12.97 (9.88, 16.29)	NA	NA	NA
Male (%)	45.87% (3085)	38.68% (200)	51.72% (765)	0.002	<0.001	<0.001
Hypertension (%)	60.90% (4096)	56.87% (294)	67.41% (997)	0.071	<0.001	<0.001
DM (%)	19.48% (1310)	23.60% (122)	20.96% (310)	0.023	0.194	0.210
CAD (%)	18.09% (1217)	31.91% (135)	20.01% (296)	<0.001	0.085	0.004

p^1^, non-obese SLD vs. control; p^2^, obese MASLD vs. control; p^3^, non-obese SLD vs. obese MASLD; NA, not applicable.

Compared to the obese MASLD group, the non-obese SLD group exhibited significantly lower BMI, RBC, HBG, Cr, UA, TG, and ALT levels, as well as a lower proportion of males and prevalence of hypertension. However, age, FBG, TC, HDL, and prevalence of CAD were significantly higher in the non-obese SLD group. No significant differences were observed in HbA1c, WBC, ANC, PLT, urea, LDL, Apo B/A1, AST, Alb, TB, or prevalence of DM between these two groups.

Compared to the control group, the non-obese SLD group showed significantly elevated levels of FBG, HbA1c, PLT, TC, TG, LDL, Apo B/A1, ALT, AST, and Alb, along with a higher prevalence of DM and CAD. While age, BMI, urea, Cr, HDL, and the proportion of males were significantly lower. There were no differences in WBC, ANC, RBC, HGB, UA, TB, or the prevalence of hypertension levels. In the comparison between the obese MASLD and control groups, BMI, FBG, HbA1c, WBC, ANC, RBC, PLT, UA, TC, TG, Apo B/A1, ALT, AST, Alb, the proportion of males, and the prevalence of hypertension and DM were significantly increased in the obese MASLD group, while age and HDL were significantly decreased. No significant differences were found in urea, Cr, LDL, TB levels, or the prevalence of DM. Notably, the prevalence of CAD in non-obese SLD was significantly higher than that in both the control group and obese MASLD group, suggesting that non-obese SLD is closely related to the occurrence of CAD.

### 3.2 Demographic and clinical characteristics of non-obese SLD patients with or without CAD in the training set

Due to the higher probability of CAD in non-obese SLD, we further explored potential risk factors for CAD in non-obese SLD. The non-obese SLD patients were randomly divided into a training set (*n* = 310) and a validation set (*n* = 207). Baseline characteristics were comparable between the two sets, except for PLT and TBIL levels (Supplementary Table 2). We further compared the clinical features of non-obese SLD patients with or without CAD in the training set (Table 2). Compared to those without CAD, non-obese SLD patients with CAD were significantly older and exhibited higher levels of FBG, HbA1c, WBC, ANC, TC, HDL, and LDL. Additionally, this group showed a higher proportion of males and a higher prevalence of DM and hypertension. No significant differences were observed in RBC, HGB, PLT, Urea, Cr, UA, TG, ALT, AST, Alb, or TBIL levels between these two groups.

**Table 2:** Demographic and clinical characteristics of non-obese SLD patients with or without CAD in the training set.

Variables (*n* (%) or median (IQR))	Without CAD (*n* = 231)	With CAD (*n* = 79)	*p-*value
Age (years)	59 (53, 65)	65 (61, 72.5)	<0.001
FBG (mmol/L)	5.53 (5.01, 6.43)	6.36 (5.39, 9.00)	<0.001
HbA1c (%)	5.80 (5.50, 6.20)	6.30 (5.75, 8.10)	<0.001
WBC (×10^9^/L)	5.91 (4.85, 6.84)	6.44 (5.39, 7.44)	0.02
ANC (×10^9^/L)	3.50 (2.73, 4.37)	4.03 (2.98, 4.92)	0.02
RBC (×10^12^/L)	4.56 (4.31, 4.89)	4.62 (4.26, 4.97)	0.663
HGB (g/L)	139 (130, 149.75)	140 (131, 154)	0.168
PLT (×10^9^/L)	226 (188.25, 263)	209 (191, 235)	0.064
Urea (mmol/L)	5.50 (4.67, 6.50)	5.79 (4.81, 7.08)	0.11
Cr (μmol/L)	61.10 (52.23, 70.15)	65.10 (54.85, 75.15)	0.121
UA (μmol/L)	337.88 (282.62, 395.82)	320.80 (265.86, 385.85)	0.124
TC (mmol/L)	5.02 (4.46, 5.40)	5.77 (4.86, 6.72)	<0.001
TG (mmol/L)	1.59 (1.13, 2.40)	1.50 (1.17, 1.85)	0.282
HDL (mmol/L)	1.14 (1.01, 1.33)	1.44 (1.25, 1.65)	<0.001
LDL (mmol/L)	2.69 (2.23, 3.11)	3.12 (2.50, 4.04)	<0.001
Apo B/A1	0.70 (0.58, 0.82)	0.67 (0.54, 0.83)	0.259
ALT (IU/L)	24.60 (18.09, 34.88)	21.65 (17.08, 29.65)	0.114
AST (IU/L)	21.97 (17.73, 26.24)	21.25 (16.64, 26.59)	0.384
Alb (g/L)	43.35 (41.08, 45.50)	41.84 (39.57, 44.93)	0.06
TBIL (μmol/L)	12.91 (10.12, 17.11)	14.14 (11.18, 17.70)	0.312
Male (%)	36.36% (84)	51.90% (41)	0.015
Hypertension (%)	51.94% (120)	69.62% (55)	0.006
DM (%)	17.75% (41)	40.51% (32)	<0.001

### 3.3 Independent risk factors of non-obese SLD patients with CAD in the training set

To determine independent risk factors for CAD in non-obese SLD patients, we conducted a multivariate logistic regression analysis. After excluding variables with multicollinearity, the factors with statistical differences between the non-obese SLD patients with or without CAD in the training set were included in the multivariate logistic regression analysis (Supplementary Table 3). The results indicated that male sex, history of DM, age, and elevated TC (with odds ratios of 3.266, 2.961, 1.079, and 2.420, respectively) were independently associated with the presence of CAD in non-obese SLD.

### 3.4 Constructing and validating a nomogram for predicting CAD in non-obese SLD patients

**Figure 1:**
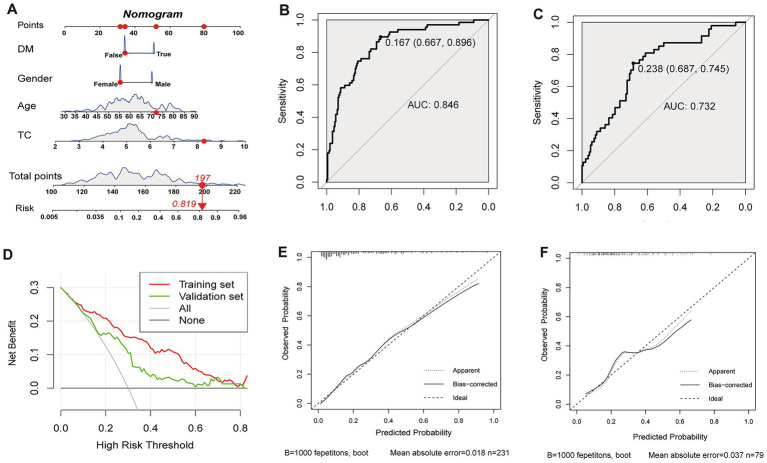
Construction and validation of a nomogram for predicting CAD in non-obese SLD patients. **(A)** A nomogram for predicting CAD in non-obese SLD in the training set. **(B)** ROC curve of the nomogram for diagnosing CAD in non-obese SLD in the training set. **(C)** ROC curve of the nomogram for diagnosing CAD in non-obese SLD in the validation set. **(D)** DCAs for the prediction of CAD in the training set and validation set. The x-axis is the threshold probability, and the y-axis is the net benefit. **(E)** The calibration curve of the prediction model in the training set. **(F)** The calibration curve of the prediction model in the validation set.

To further predict the risk of CAD in patients with non-obese SLD in the clinic, we constructed a nomogram based on the results of multivariate logistic regression analysis. In this nomogram, each variable was assigned a score on the points scale. The total score was calculated by summing these individual scores and was then projected onto the total points scale to estimate the probability of CAD (Figure 1A).

The AUC of the logistic regression model in the training set was 0.846, and the cutoff value, specificity, and sensitivity were 0.167, 66.7, and 89.6%, respectively (Figure 1B). The accuracy, positive predictive value (PPV), and negative predictive value (NPV) of this model were 74.5, 47.6, and 95.0%, respectively (Supplementary Table 4). Next, the model was evaluated in the validation set. The AUC of the logistic regression model was 0.732, and the cutoff value, specificity, and sensitivity were 0.238, 68.7, and 74.5%, respectively (Figure 1C). The accuracy, PPV, and NPV were 70.2, 45.5, and 88.5%, respectively (Supplementary Table 4). Both DCAs in the training set and validation set lie above the “All” and “None” curves, indicating significant net clinical benefit (Figure 1D). Both calibration curves in both sets were close to the baselines, demonstrating good consistency between predicted and actual CAD probabilities (Figures 1E,F).

### 3.5 Identification of DEGs in the non-obese SLD dataset and CAD dataset

**Figure 2:**
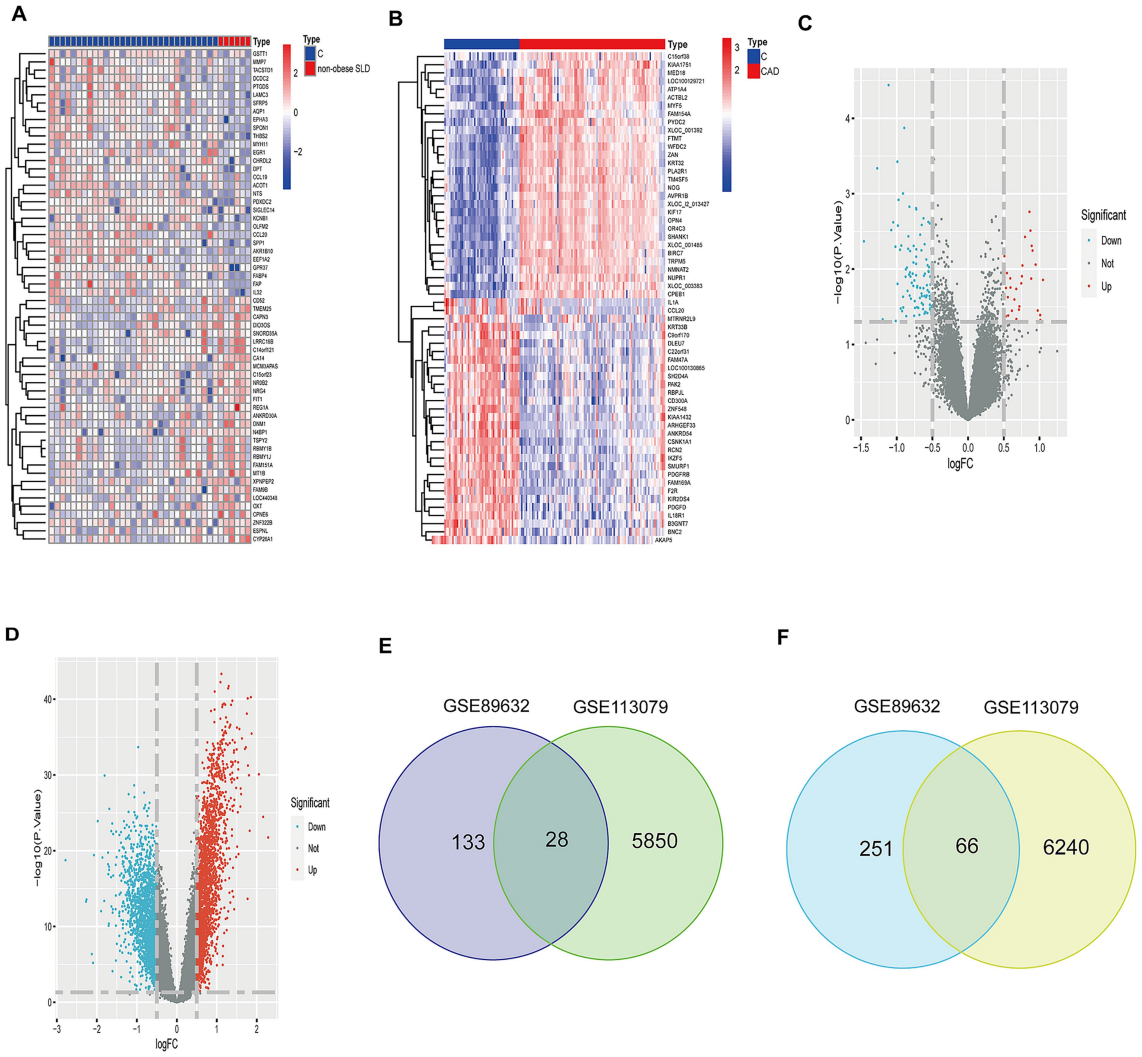
Screening of DEGs in non-obese SLD dataset (GSE98632) and CAD dataset (GSE113079). **(A)** Heatmap of the top 60 DEGs for the non-obese SLD dataset. **(B)** Heatmap of the top 60 DEGs for the CAD dataset. **(C)** The volcano plots indicate the DEGs for the non-obese SLD dataset. **(D)** The volcano plots indicate the DEGs for the CAD dataset. Vertical line indicated log_2_ FC and horizontal line indicated -log_10_ (*p*-values). **(E)** Venn diagram of overlapping upregulated DEGs in the non-obese SLD dataset and CAD dataset. **(F)** Venn diagram of overlapping downregulated DEGs in the non-obese SLD dataset and CAD dataset.

Based on the established significant association between non-obese SLD and CAD, we sought to investigate potential shared hub genes involved in their interaction. To this end, we analyzed two relevant datasets: the non-obese SLD dataset (GSE89632, liver tissue expression profiles) and the CAD dataset (GSE113079, peripheral blood mononuclear cell expression profiles). There were 477 DEGs (161 upregulated genes and 316 downregulated genes) in the non-obese SLD dataset and 4,622 DEGs (2089 upregulated genes and 2,533 downregulated genes) in the CAD dataset. The expression patterns of the top 60 significant DEGs (ranked by *p*-value) were visualized in heatmaps (Figures 2A,B), and the overall distribution of DEGs was presented in volcano plots (Figures 2C,D). There were 28 overlapping upregulated DEGs and 66 overlapping downregulated DEGs between the non-obese SLD and CAD datasets, as shown in the Venn diagram in Figures 2E,F.

### 3.6 Construction of the PPI network and identification of hub genes

**Figure 3:**
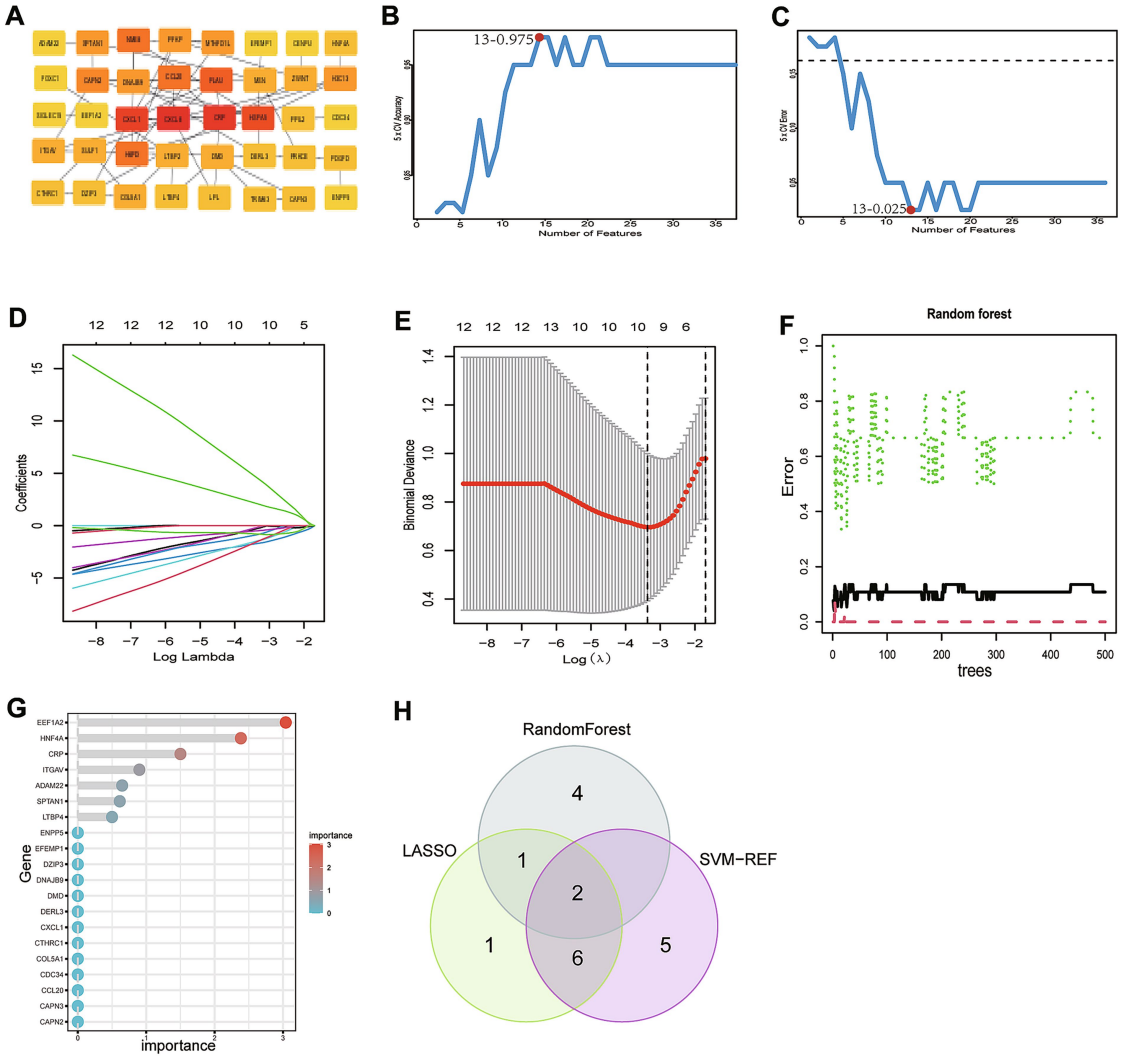
PPI network for DEGs and machine-learning algorithm screening for optimal core genes of CAD in non-obese SLD. **(A)** PPI network of the top 40 genes with the highest node scores from the MCC algorithm. **(B)** The accuracy was the highest when the feature was 13 for SVM-RFE analysis after 100 folds. **(C)** The error of the classifier was the smallest when the feature was 13 for SVM-RFE analysis after 100 folds. **(D,E)** The lowest binomial deviance of the LASSO regression curve corresponds to the most suitable candidate number of genes (*n* = 10). **(F)** The relationship between the number of trees and the error rate of the RF. **(G)** Ranking of genes based on the importance score calculated from RF. **(H)** Venn diagram shows that two core genes are screened by three machine-learning algorithms.

After merging the DEGs that were simultaneously upregulated or downregulated in both datasets, we obtained 94 common genes. Subsequently, the most significant genes were recognized by the plug-in CytoHubba APP of Cytoscape, and the top 40 genes with the highest node scores from the MCC algorithm were considered hub genes (Figure 3A).

### 3.7 Integrating SVM-RFE, LASSO, and RF for screening the core genes

In order to further narrow down the candidate biomarkers, we further performed three machine-learning algorithms (SVM-RFE, LASSO, and RF) for the 40 DEGs obtained above. We performed an in-depth screening by SVM-RFE with 100 folds, and the results showed that when 13 genes were included, the classifier had the smallest error and the highest accuracy (Figures 3B,C). We further screened the core genes by LASSO regression. We identified 10 candidate genes with the lowest binomial deviance (Figures 3D,E). Furthermore, we determined seven genes using the RF algorithm with an importance score > 0 (Figures 3F,G). A Venn diagram showed there were two core genes named hepatocyte nuclear factor 4α (HNF4A) and latent transforming growth factor *β* (TGFβ) binding protein 4 (LTBP4) that were crossed among these three machine learning algorithms (Figure 3H). These two genes were selected for subsequent analysis.

### 3.8 Exploring the expression and predictive values of the core genes based on the datasets

**Figure 4:**
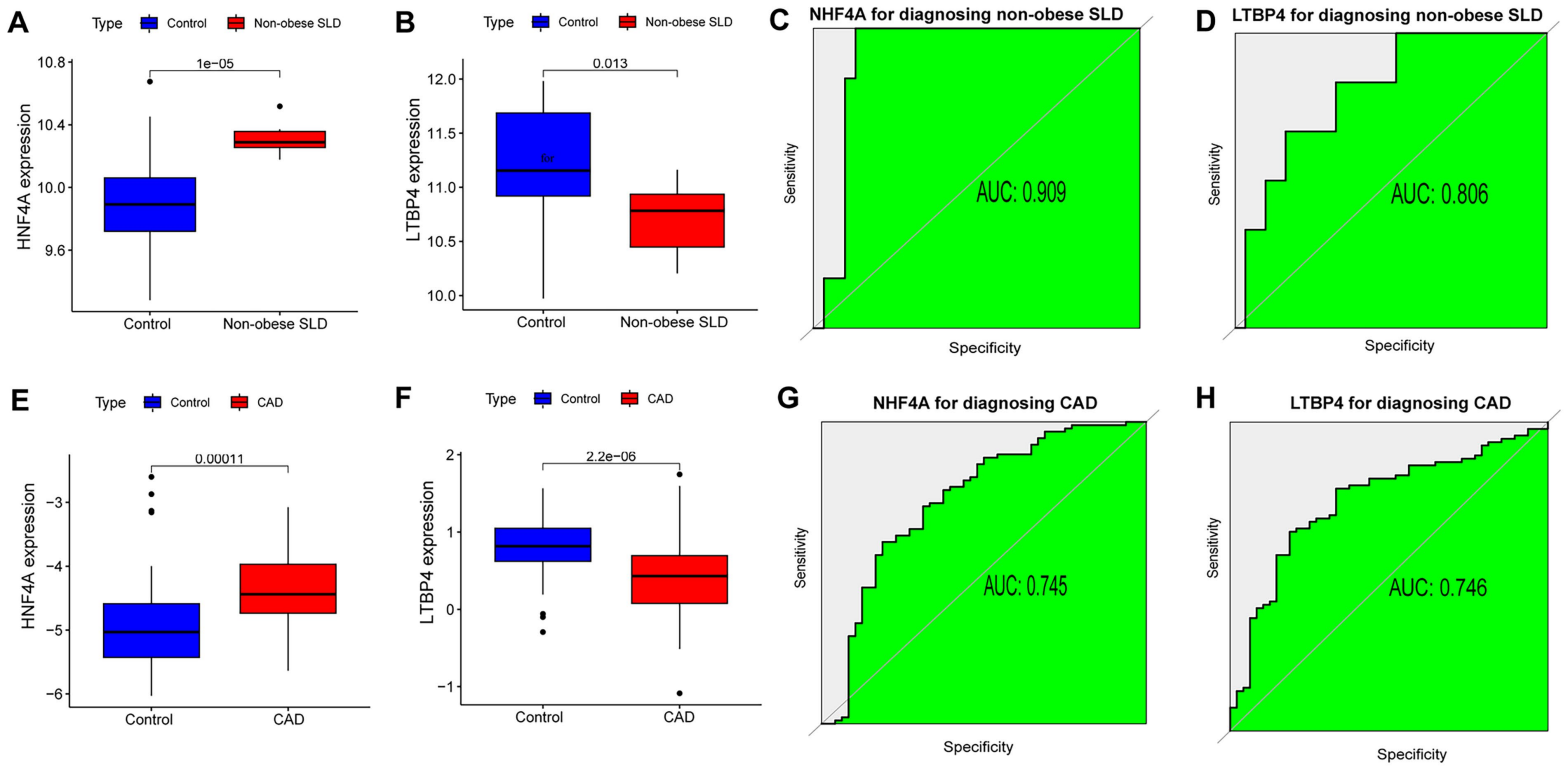
Expression patterns and diagnostic efficacy of core genes in non-obese SLD and CAD based on the datasets. Expression levels of HNF4A **(A)** and LTBP4 **(B)** in non-obese SLD. The ROC curves of HNF4A **(C)** and LTBP4 **(D)** for diagnosing non-obese SLD. Expression levels of HNF4A **(E)** and LTBP4 **(F)** in CAD. The ROC curves of HNF4A **(G)** and LTBP4 **(H)** for diagnosing CAD.

The expression level of HNF4A was significantly higher (Figure 4A), while that of LTBP4 was significantly lower (Figure 4B) in the non-obese SLD group compared to healthy individuals in GSE89632. The AUCs of HNF4A and LTBP4 for diagnosing non-obese SLD were 0.909 and 0.806 (Figures 4C,D). The expression level of HNF4A was significantly higher (Figure 4E), while LTBP4 was significantly lower (Figure 4F) in the CAD group compared to healthy individuals in GSE113079. The AUCs of HNF4A and LTBP4 for diagnosing CAD were 0.745 and 0.746 (Figures 4G,H). These results confirmed that both core genes possessed significant predictive value for both diseases.

## 4 Discussion

SLD is generally considered to be associated with obesity, but it can also occur in individuals with BMI < 25 kg/m^2^, a phenotype termed “non-obese SLD.” A meta-analysis reported that non-obese SLD accounts for 20% of the non-obese population (26). According to previous reports, liver-related mortality (26) and all-cause mortality (19) are significantly higher in non-obese SLD patients compared to their obese counterparts. These findings underscore the high prevalence and poor prognosis of this condition; therefore, increased clinical attention is warranted for this specific population.

Non-obese SLD accounts for 36–45% of the global SLD population (26–29). In our retrospective study, non-obese SLD accounted for 25.9% of the total SLD patients, which is lower than the reported value in previous investigations. This discrepancy may be that most subjects in our study were suspected of CAD, and obesity is an explicit risk factor for CAD. Consequently, our cohort likely included a disproportionately higher number of obese individuals. Compared to obese MASLD patients, non-obese SLD patients exhibited lower levels of FBG, UA, ALT, and TG, and a lower prevalence of hypertension, generally indicating a more favorable metabolic profile. However, the non-obese SLD group also showed high-risk characteristics, such as significantly increased TC levels and a higher prevalence of CAD. These findings suggest that the increased risk of CAD in non-obese SLD patients may be driven by dysregulated cholesterol metabolism. The association between abnormal cholesterol levels and CAD is well-documented. A study involving 4,467,942 veterans concluded that a lower serum cholesterol level was associated with a reduced risk of CAD (30). Another study reached similar conclusions that an elevated level of total cholesterol indicates an increased mortality risk of CAD (31, 32). Therefore, we hypothesize that the pathogenesis of CAD in non-obese SLD involves aberrant cholesterol metabolism, a mechanism that warrants further investigation.

In this study, a logistic regression model for predicting CAD in non-obese SLD was created with available laboratory parameters. A nomogram was constructed incorporating four independent predictors: history of DM, male sex, age, and TC. To verify the clinical usability of this model, calibration curves and DCA were performed in the training set and the validation set. We found that the nomogram we built was highly effective in predicting CAD in non-obese SLD. To the best of our knowledge, this is the first study to use such a comprehensive approach to construct a predictive model for CAD in non-obese SLD patients. The performance of this model was stable in both the training set and validation set. In addition, our nomogram offers distinct advantages over traditional criteria. Nomogram is a user-friendly tool that could guide clinical decision-making for doctors, as it can transform a complex statistical prediction model into a single numerical estimate based on different event probabilities. By summing the scores of different predictive variables, clinicians can quickly estimate an individual patient’s risk without complex calculations. By integrating multiple clinical variables, the nomogram provides a more precise prediction compared to single-factor assessments.

In this study, HNF4A and LTBP4 were selected as core genes based on an integrated bioinformatic analysis of the non-obese SLD dataset (GSE89632) and CAD (GSE113079) dataset. We also found that HNF4A and LTBP4 showed good accuracy of diagnosis for both non-obese SLD and CAD, with AUCs exceeding 0.7.

HNF4A is a pivotal liver transcription factor belonging to the nuclear receptor superfamily, highly expressed in the liver, intestine, kidney, and pancreas. The expression of HNF4A in MASLD remains controversial. Multiple studies have shown that HNF4A was overexpressed in the liver tissues of mice with a high-sugar diet and MASLD patients (33, 34), whereas some other studies have demonstrated that HNF4A was significantly reduced in high-fat diet-fed diabetic mice and MASH patients (35, 36). Our analysis revealed elevated HNF4A levels in both CAD and non-obese SLD patients. It plays a crucial role in the regulation of glucose and lipid metabolisms (37, 38). Thymiakou et al. (39) found that liver steatosis and liver triglyceride content increased, whereas the concentration of serum total cholesterol, HDL cholesterol, and triglycerides decreased in mice with liver-specific ablation of HNF4A. HNF4A deficiency promotes lipid accumulation within the liver while lowering cholesterol and triglyceride levels within the serum (40). In addition, our data indicated higher serum cholesterol levels in the non-obese SLD cohort. Thus, we hypothesized that the overexpression of HNF4A might influence hepatic fat deposition by disrupting cholesterol metabolism, thereby contributing to CAD in non-obese individuals. However, the specific mechanism remains unclear. Further investigation into the structural and functional properties of HNF4A may uncover novel mechanisms of systemic cholesterol homeostasis, offering potential therapeutic strategies.

The second core gene identified was LTBP4, which belongs to the LTBP family. LTBP4 can maintain the latent (inactive) state of TGF-*β* in the extracellular matrix by binding to TGF-β (41). Therefore, a decrease in LTBP4 expression leads to aberrant TGF-β activity. TGF-β plays a role in controlling cell growth and differentiation, and is involved in the pathogenesis of MASLD and CAD (42, 43). Several studies have found that the expression of TGF-β increases in NASH livers and stimulates the development of fibrosis (44–46). Particularly, Ling Yang et al. reported that TGF-β signaling promotes hepatocyte steatosis, inflammatory cell infiltration, inflammatory cytokine production, and fibrosis, and participates in the pathogenesis of steatohepatitis by regulating cell death and lipid metabolism in the MASLD mouse model (46). In the context of CAD, patients with severe coronary artery obstruction (>50% stenosis in multiple major vessel lumens) have significantly elevated levels of active TGF-β1 compared to healthy individuals or those with mild obstruction (47, 48). In our study, LTBP4 was downregulated in both non-obese SLD and CAD. Therefore, we hypothesized that the inhibition of LTBP4 aggravated non-obese SLD and CAD by unleashing the TGF-β signaling pathway. Future research on LTBP4 may help to develop new therapeutic strategies, particularly for patients with comorbid non-obese SLD and CAD.

Our study has several limitations. First, as a retrospective single-center study, potential selection bias is inevitably present in the prediction model for CAD in non-obese SLD patients. Therefore, the conclusions drawn from this study need to be validated through large-scale, multi-center cohorts. Second, the core genes were identified from public datasets with limited sample sizes. Although we interpreted the research results based on comprehensive bioinformatics analysis, *in vitro* and *in vivo* experiments, experimental verification is essential. Nevertheless, it cannot be denied that our analysis possesses multiple notable strengths. Thirdly, to ensure that no critical biological signals were overlooked, we adopted more inclusive criteria during the initial screening phase. To remedy this issue, these initial candidates were subsequently refined through a rigorous, multi-layered bioinformatic pipeline—integrating PPI network analysis with three distinct machine learning algorithms. This ‘consensus-based’ strategy effectively filtered out noise and isolated highly credible core genes, thereby ensuring the robustness and validity of our conclusions. To our knowledge, this is the first study to construct a diagnostic model for CAD in non-obese SLD using common clinical indicators. This model may obviate the need for invasive procedures, enabling early risk stratification. We also utilized comprehensive bioinformatics analysis and machine learning to uncover the molecular link between non-obese SLD and CAD. In addition, for the diagnosis of CAD, previous studies mostly used clinical indicators to calculate risk scores to infer the presence of CAD, resulting in a lack of accuracy in diagnosis. CCTA is a well-validated non-invasive method. In our study, we used CCTA for evaluating coronary artery disease, thereby significantly improving diagnostic accuracy. We believe that this study contributes to elucidating new pathogenic mechanisms and may provide new therapeutic strategies for patients with CAD and non-obese SLD.

## 5 Conclusion

Non-obese SLD patients face a higher risk of CAD compared to the general population or obese patients. Our study developed a novel diagnostic model incorporating gender, age, history of DM, and TC to predict CAD in non-obese SLD patients. This model demonstrated robust predictive performance in both training (AUC = 0.846) and validation (AUC = 0.732) sets. Furthermore, comprehensive bioinformatics analysis and machine learning algorithms identified two core genes, HNF4A and LTBP4, which were associated with both conditions and possessed significant diagnostic value. These findings suggest potential pathogenic mechanisms involving HNF4A and LTBP4, warranting further molecular validation to explore their therapeutic potential.

## Data Availability

The raw data supporting the conclusions of this article will be made available by the authors, without undue reservation.

## Footnotes

1
http://www.ncbi.nlm.nih.gov/geo

2
http://string-db.org/

3
https://apps.cytoscape.org/apps/cytohubba

## Glossary

Alb: albumin
ALT: alanine aminotransferase
ANC: absolute neutrophil count
ApoB/A1: apolipoprotein B/ Apolipoprotein A1
AST: aspartate aminotransferase
AUC: the area under the receiver operating characteristics curve
BMI: body mass index
CAD: coronary artery disease
CCTA: coronary computed tomography angiography
CI: confidence interval
Cr: creatinine
DCA: decision curve analysis
DEGs: differentially expressed genes
DM: diabetes
FBG: fasting blood glucose
GEO: Gene Expression Omnibus
HGB: hemoglobin
HbA1c: hemoglobin A1c
HDL-C: high-density lipoprotein cholesterol
LASSO: the least absolute shrinkage and selection operator
LDL-C: low-density lipoprotein cholesterol
MCC: maximal clique centrality
NAFLD: non-alcoholic fatty liver disease
NPV: negative predictive value
OR: odds ratio
PLT: platelet count
PPI: protein–protein interaction
PPV: positive predictive value
RBC: red blood cell
RDW: red cell distribution width
RF: random forest
ROC: receiver operating characteristic
SVM-RFE: support vector machine-recursive feature elimination
TBIL: total bilirubin
TC: total cholesterol
TG: triglyceride
UA: uric acid
WBC: white blood cell
SLD: steatotic liver disease
MASLD: metabolic dysfunction-associated steatotic liver disease
