# Supplementary material for: Exploring the prediction model and core genes for coronary artery disease in non-obese steatotic liver disease patients
Source: Front Med (Lausanne). 2026 Feb 9;13:1709412. doi: 10.3389/fmed.2026.1709412 (PMC12926503; doi:10.3389/fmed.2026.1709412)
Supplement: Supplementary file 1 [file Data_Sheet_1.pdf]

Supplementary Table 1. Detailed information of GEO datasets used in this study

Data sets	Platform	Disease type	Control sample size	Disease sample size
GSE89632	GPL14951	non-obese SLD	31	6
GSE113079	GPL20115	CAD	48	93

Supplementary Table 2. Comparision of demographic and clinical characteristics of patients with non-obese SLD in the training and validation set.

Variables ((n (%) or median (IQR))	Training set (n=310)	Validation set (n=207)	*p* value
Age (years)	61 (54, 67.25)	61 (55, 67)	0.300
BMI (kg/m^2)	23.94 (22.86, 24.61)	23.88 (22.86, 24.53)	0.401
FBG (mmol/L)	5.70 (5.14, 7.10)	6.00 (5.25, 7.10)	0.136
HbA1c (%)	5.90 (5.60, 6.70)	5.90 (5.60, 6.30)	0.622
WBC (×10^9/L)	6.05 (4.99, 7.02)	6.29 (5.34, 7.43)	0.099
ANC (×10^9/L)	3.66 (2.86, 4.58)	3.71 (3.02, 4.75)	0.156
RBC (×10^{12}/L)	4.60 (4.29, 4.91)	4.55 (4.33, 4.86)	0.705
HGB (g/L)	139 (130, 151)	138 (130, 148)	0.162
PLT (×10^9/L)	220.50 (187.00, 261.25)	233.00 (201.00, 270.50)	0.037
Urea (mmol/L)	5.60 (4.70, 6.60)	5.50 (4.80, 6.47)	0.956
Cr (μ mol/L)	61.10 (51.78, 71.53)	61.73 (52.24, 71.63)	0.823
UA (μ mol/L)	332.40 (275.47, 392.55)	335.20 (289.05, 403.75)	0.334
TC (mmol/L)	5.12 (4.54, 5.65)	5.04 (4.44, 5.68)	0.593
TG (mmol/L)	1.59 (1.45, 2.31)	1.51 (1.19, 2.33)	0.763
HDL (mmol/L)	1.16 (1.04, 1.43)	1.16 (1.06, 1.43)	0.831
LDL (mmol/L)	2.76 (2.21, 3.28)	2.76 (2.15, 3.31)	0.657
Apo B/A1	0.69 (0.57, 0.82)	0.68 (0.57, 0.81)	0.563
ALT (IU/L)	24.40 (18.10, 32.66)	22.36 (17.37, 32.59)	0.464
AST (IU/L)	21.97 (17.40, 26.60)	20.93 (17.75, 26.23)	0.707
Alb (g/L)	43.22 (40.79, 45.39)	42.74 (40.80, 45.48)	0.732
TBIL (μmol/L)	13.25 (9.94, 16.92)	12.06 (9.39, 15.10)	0.004
Male (%)	39.43% (125)	37.50% (75)	0.349
Hypertension (%)	55.21% (175)	59.50% (119)	0.816
DM (%)	23.03% (73)	24.50% (49)	0.973

Supplementary Table 3. Independent risk factors of non-obese SLD patients with CAD in the training set.

	OR value	(95% CI)	*p* value
Male (%)	3.566	1.813-7.015	<0.001
Hypertension (%)	1.248	0.641-2.430	0.515
DM (%)	2.961	0.170-0.670	0.002
Age (years)	1.079	1.043-1.116	<0.001
WBC ($\times 10^9$/L)	1.201	0.993-1.452	0.509
TC (mmol/L)	2.420	1.492-5.874	<0.001

Supplementary Table 4. Evaluation indexes of prediction model of CAD in non-obese SLD patients in the training set and validation set.

	AUC (95% CI)	threshold	specificity (%)	sensitivity (%)	accuracy (%)	PPV (%)	NPV (%)
training set	0.846 (0.667-0.896)	0.167	66.7	89.6	74.5	47.6	95.0
validation set	0.732 (0.687-0.745)	0.238	68.7	74.5	70.2	45.5	88.5
